# An Unsuspected Case of Euglycemic Diabetic Ketoacidosis With Twists

**DOI:** 10.7759/cureus.24016

**Published:** 2022-04-10

**Authors:** Cyra-Yoonsun Kang, Parnia Khamooshi, Viviana Reyes Pinzon

**Affiliations:** 1 Internal Medicine, John H. Stroger, Jr. Hospital of Cook County, Chicago, USA

**Keywords:** sglt-2 inhibitor, swallowed foreign body, latent autoimmune diabetes in adults (lada), euglycemic, diabetic ketoacidosis (dka)

## Abstract

Euglycemic diabetic ketoacidosis (EDKA) is defined by acidosis, ketones in serum and urine, and a high anion gap (AG) with a normal glucose level. Sodium-glucose cotransporter-2 (SGLT2) inhibitor has become one of the most common causes of EDKA. We present one of the lowest presenting blood glucose levels for the EDKA in the setting of SGLT2 inhibitor use. A 34-year-old female with a two-year history of type 2 diabetes mellitus (T2DM) presented after accidental ingestion of a metal clip and lack of oral intake for 28 hours. She reported a recent intentional weight loss of 60 lbs. She takes metformin 1000 mg twice daily and empagliflozin 25 mg daily. An urgent endoscopy was planned in the intensive care unit given the proximal location of the foreign body. The basic metabolic profile after the procedure demonstrated a glucose level of 75 mg/dL, bicarbonate level of 11 mmol/L, and AG of 17 mmol/L. The venous pH was 7.27 with a partial pressure of carbon dioxide of 30 mmHg. The urinalysis showed a glucose level greater than 500 μmol/L with a ketone level of 80 μmol/L. The blood and urine toxicology screening results were unremarkable. The patient was treated for EDKA with the administration of intravenous (IV) dextrose 5% in water with subsequent initiation of IV insulin. The assessment of her insulin reserve revealed a low C-peptide of 0.36 ng/mL, a high glutamic acid decarboxylase level greater than 250 IU/mL, and high zinc transporter 8 (ZnT8) antibodies of 42 U/mL, consistent with an undiagnosed transition to latent autoimmune diabetes in adults (LADA). The blood glucose levels for previously reported cases remain between 90 and 250 mg/dL. In this case, the combination of a low carbohydrate diet and prolonged starvation may have led to the impressively low glucose. Additionally, the SGLT2 inhibitor use in patients with LADA serves as one of the known risk factors for EDKA. EDKA poses a diagnostic challenge, especially in the ICU setting where there exists a myriad of causes for high AG metabolic acidosis. Additionally, many of the ICU patients are in a ketotic state brought on by prolonged starvation. Therefore, prompt diagnosis and treatment for EDKA require careful history taking and complete investigation for other causes of high AG metabolic acidosis.

## Introduction

High anion gap (AG) metabolic acidosis serves as one of the most common acid-base disturbances found in patients in the intensive care unit (ICU) setting. Prompt exploration of the possible etiologies is necessary for treatment to prevent potentially life-threatening outcomes. Among the etiologies, diabetic ketoacidosis (DKA) is unlikely to be missed in the context of a clear history of diabetes and the pathognomic laboratory values. Typically, DKA is defined by acidosis, ketones in serum and urine, and a high AG with blood glucose commonly above 250 mg/dl. However, in the case of euglycemic DKA (EDKA), the blood glucose level can be lower than expected as carbohydrate deficit plays a pivotal role in the keto-acidotic state [1]. We report one of the lowest presenting blood glucose levels among the cases of EDKA associated with the sodium-glucose cotransporter-2 (SGLT2) inhibitor use and exacerbated by starvation and weight loss from a low carbohydrate diet.

## Case presentation

A 34-year-old female with a past medical history of type 2 diabetes mellitus (T2DM) diagnosed at age 32 presented after accidental ingestion of a metal clip. She thought a small part of the blender fell off and went into her smoothie. She endorsed a lack of oral intake for 28 hours due to a sore throat and foreign body sensation in the upper chest when she swallows. She reported being diagnosed with T2DM two years prior at an outside hospital when she presented for a regular check-up. She stated that besides the hemoglobin A1c level, no other workup was performed. She was initiated on metformin 500 mg twice daily. In the past year, the dose was increased to 1000 mg twice daily. Most recently, due to the suboptimal control, empagliflozin 25 mg daily was added. She endorsed 20 kg weight loss over the course of two years since the diagnosis. Her body mass index changed from 29 kg/m^2^ to 20.7 kg/m^2^.

The last dose of empagliflozin was taken 20 hours prior to the presentation. Chest CT with IV contrast confirmed a sharp linear metallic density within the proximal esophagus without any evidence of perforation. Subsequently, an urgent endoscopy was performed in the intensive care unit given the limited availability of operating rooms overnight. The foreign body was retrieved without any complications.

In the following morning, she remained asymptomatic. However, the basic metabolic profile (Table 1) demonstrated an AG of 17 mmol/L, while the glucose level was at 75 mg/dL. The potassium level and the bicarbonate level were at 5.6 mmol/L and 11 mmol/L, respectively. The venous pH was 7.27 with a partial pressure of carbon dioxide (PCO2) of 30 mmHg. The urinalysis showed a urinary glucose level greater than 500 μmol/L with a ketone level of 80 μmol/L. Other causes of high AG metabolic acidosis were excluded by unremarkable extended blood and urine toxicology screening.

**Table 1:** Initial basic metabolic panel

Laboratory investigations	Reference values	Values
Sodium	136-145 mEq/L	136 mEq/L
Chloride	98-106 mEq/L	108 mEq/L
Potassium	3.5-5 mEq/L	5.6 mEq/L
Anion gap	7-13 mEq/L	17 mEq/L
Carbon dioxide	23-29 mEq/L	15 mEq/L
Creatinine	M: 0.5-1.30, F: 0.4-1.2 mg/dL	0.6 mg/dL
Blood urea nitrogen	5-25 mg/dL	10 mg/dL
Glucose	70-99 mg/dL	75 mg/dL
Albumin	3.4-5.4 g/dL	4.2 g/dL

The patient was treated for SGLT2 inhibitor-induced EDKA with the administration of IV dextrose 5% in water. Once the serum glucose level had reached 115 mmol/L, continuous IV insulin therapy at 1 unit/hour was initiated. The AG normalized 10 hours after the diagnosis of DKA. Her serum glucose remained below 140 mmol/L (Table 2). The assessment of her insulin reserve (Table 3) revealed a low C-peptide of 0.36 ng/mL alongside a high glutamic acid decarboxylase level of greater than 250 IU/mL and high zinc transporter 8 (ZnT8) antibodies of 42 U/mL. Based on her diabetes mellitus course, age, and lab findings, she was diagnosed with latent autoimmune diabetes in adults (LADA).

**Table 2:** Post-treatment basic metabolic panel

Laboratory investigations	Reference values	Values
Sodium	136-145 mEq/L	137 mEq/L
Chloride	98-106 mEq/L	109 mEq/L
Potassium	3.5-5 mEq/L	4.1 mEq/L
Anion gap	7-13 mEq/L	11 mEq/L
Carbon dioxide	23 - 29 mEq/L	17 mEq/L
Creatinine	M: 0.5-1.30, F: 0.4-1.2 mg/dL	0.6 mg/dL
Blood urea nitrogen	5-25 mg/dL	7 mg/dL
Glucose	70-99 mg/dL	129 mg/dL

**Table 3:** Anti-insulin antibody panel

Laboratory investigations	Reference values	Values
C-peptide	0.5-2.0 ng/mL	0.36 ng/mL
Glutamic acid decarboxylase	<5 IU/mL	250 IU/mL
Zinc transporter 8 antibodies	Age >/= 30 years, <10 U/mL	42 U/mL

## Discussion

The diagnosis of EDKA takes a discerning eye due to the absence of hyperglycemia typically seen in DKA. The patient in this case also did not present with typical symptoms of EDKA. However, she had multiple risk factors including SGLT2 inhibitor use, starvation, a low carbohydrate diet, and a missed diagnosis of LADA. The composite of the risk factors leading to one of the lowest reported glucose levels in EDKA renders this case unique.

SGLT2 inhibitors promote glucosuria leading to decreased plasma glucose level, which promotes glucagon production and discourages insulin release thereby inducing DKA with low blood glucose [1]. Reported median glucose levels range between 211 and 328 mg/dL [2,3]. DKA tends to have precipitating factors that cause stress in the body, such as preoperative and postoperative settings, sepsis, dehydration, and starvation [3-5]. The various forms of stress may lead to decreased oral intake, which lowers blood glucose levels, especially in the setting of concurrent SGLT2 inhibitor use. The sustained low glucose level results in ketosis, which can be exacerbated by increased ketone resorption by SGLT2 inhibitors.

Our patient also adhered to a strict low-carbohydrate diet with only 20% of her meals consisting of carbohydrates. Her low-carbohydrate diet in the setting of continuous SGLT2 inhibitors use increased glucagon secretion while suppressing serum glucose levels. Low-carbohydrate diet has been known to cause EDKA as evidenced by previously reported cases [4,6-8]. The ketotic state due to the low carbohydrate intake can exaggerate the actions of SGLT2 inhibitors in upregulating glucagon release and suppressing insulin release while promoting glucosuria [1]. This potentially harmful combination explains such low glucose levels in our patient.

Prior to the presentation, our patient also endorsed a significant weight loss. Therefore, our patient's age and low BMI prompted the evaluation for LADA, although she did not endorse any family history of LADA [9]. Patients with LADA are thought to have a progressive course of autoimmune diabetes, which renders the diagnosis difficult [10]. The broad characteristics of LADA include ages between 30 and 50 years, personal or family history of autoimmunity, BMI less than 25 kg/m^2^, and acute symptoms at onset. At the time of her T2DM diagnosis at the age of 30, she did not appear to have any features to suggest LADA except for her age [11]. SGLT2 was prescribed based on the diagnosis of T2DM without the proper screening for LADA. Due to the risk of EDKA, the use of SGLT2 in LADA remains off-label and discouraged by many experts as close monitoring in the outpatient setting is difficult [12]. Notably, a previous study of 96 case reports identified seven cases of SGLT2 inhibitor-induced EDKA uncovering undiagnosed LADA [13]. Therefore, diabetic patients on SGLT2 inhibitors who present with ketoacidosis should undergo careful evaluation for EDKA and be reviewed for common features associated with LADA.

## Conclusions

EDKA poses a diagnostic challenge, especially in the ICU setting where there exists a myriad of causes for high AG metabolic acidosis. In this case, the cause of EDKA initially was not apparent. However, it was later discovered that she had multiple risk factors for EDKA. Therefore, prompt diagnosis and treatment for EDKA require careful history taking and complete investigation to rule out other causes of high AG metabolic acidosis. Additionally, a review of diabetes history to consider a missed diagnosis of LADA seems pertinent in individuals with low BMI and ages between 30 and 50 years.
